# Kai 1 and Kai 2: Characterization of these dog erythrocyte antigens by monoclonal antibodies

**DOI:** 10.1371/journal.pone.0179932

**Published:** 2017-06-29

**Authors:** Jae Ho Lee, Urs Giger, Hee Young Kim

**Affiliations:** 1 KABB Bio Laboratory, Department of Physiology, College of Korean Medicine, Daegu Haany University, Daegu, South Korea; 2 Section of Medical Genetics, School of Veterinary Medicine, University of Pennsylvania, Philadelphia, Pennsylvania, United States of America; INSERM1163, FRANCE

## Abstract

*Dog Erythrocyte Antigens (DEA)* have thus far been found by sensitizing dogs with canine allogeneic blood and are clinically important regarding blood transfusion incompatibilities, but remain poorly defined. The goals of this study were to discover and characterize two *DEAs*, named as *Kai 1* and *Kai 2*. The monoclonal antibodies were produced by mouse hybridoma techniques and examined by ELISA isotyping, immunoblotting, and affinity chromatography. Canine blood samples were typed and the development of alloantibodies was examined in transfused dogs. The monoclonal *Kai 1* and *Kai 2* antibodies were isotyped as IgM kappa and IgG3 lamda, respectively, and identified two different erythrocyte membrane proteins of 200 kDa and 80 kDa in molecular weights, respectively. Either *Kai 1* or *Kai 2* can be expressed but not both in an individual dog. There were no naturally occurring anti-*Kai 1* or *Kai 2* alloantibodies. In addition, *Kai 1-* and/or *Kai 2-* dogs developed *Kai 1* and *Kai 2* alloantibodies, respectively, when transfused with mismatched blood. This is the first discovery of canine blood types by screening monoclonal antibodies. *Kai 1* and *Kai 2* are novel blood types which can induce anti-*Kai 1* or anti-*Kai 2* alloantibodies when *Kai 1-* and/or *Kai 2-* dogs are transfused with *Kai 1+* or *Kai 2+* blood. These canine blood types may explain some of the blood incompatibilities and transfusion reactions observed in dogs in clinical practice.

## Introduction

Dogs have been used in early xenotransfusions to humans as well as animal models to characterize transfusion reactions [1]. In veterinary clinical practice, when anemic or bleeding dogs are transfused, blood type incompatibilities have been documented based upon hemolytic transfusion reactions and incompatible agglutination crossmatch test results [2,3]. Based upon experimentally sensitizing dogs with canine blood transfusions, eight *Dog Erythrocyte Antigens (DEA)* were classified with polyclonal alloantibodies by an international committee in 1974, but currently only reagents for *DEA 1*, *DEA 3*, *DEA 4*, and *DEA 7* are commercially available. Dogs are either positive or negative for a particular blood type except for the *DEA 1* blood group system where dogs were classified to be *DEA 1*.*1+* or *DEA 1*.*1-* and those *DEA 1*.*1-* could be *DEA 1*.*2+* or *DEA 1*.*2-* [3,4]. Recent flow cytometry and strip kit typing studies with monoclonal antibodies reveal that dogs are either *DEA 1-* or weakly to strongly *DEA 1+* [5], but the biochemical and molecular basis remains elusive for *DEA 1* and other DEAs.

Additionally, non-officially classified red blood cell (RBC) antigens have been identified in dogs, including *Dal* (Dalmatian) [6,7] and *Shigeta* dog blood types [8]. Furthermore, dogs with acute hemolytic transfusion reactions following a second transfusion [2,9] as well as major crossmatch incompatibilities after a first transfusion have been reported [3], indicating the development of alloantibodies to unknown RBC antigens different from conventional *DEAs*.

By using mouse hybridoma technique, we obtained two monoclonal antibodies against two erythrocytic membrane antigens which did not coexist in any dogs but could both be absent. The two monoclonal antibodies (mAb) and corresponding antigens, named *Kai 1* and *Kai* 2 (meaning ‘dog’ in Korean), were biochemically characterized by enzyme-linked immunosorbent assay (ELISA), immunoblot and affinity chromatography. Their antigenicity was examined in dogs after receiving inadvertently mismatched transfusions.

## Materials and method

### Animals and samples

Female mice (BALB/c, 6 weeks old, Daehan Animal, Seoul, South Korea) were used. All mice had free access to food and water and were maintained on a 12 hr light-dark cycle. Ethylenediaminetetraacetic acid (EDTA) blood samples from non-transfused and previously transfused dogs were obtained from small animal hospitals and Korea Animal Blood Bank (KABB, Sokchosi, South Korea). We obtained written consent from the dog owners to allow for experimental use of the laboratory results of blood samples collected during medical treatment (S1 file). All animal procedures were approved by the Institutional Animal Care and Use Committee at the Daegu Haany University.

### Screening and production of monoclonal antibodies against canine blood: Kai-1 and Kai-2

Hybridomas producing monoclonal antibodies were made according to the previous method [10,11] with slight modifications. In brief, mice (n = 6) were sensitized with canine RBC from two Korean Mastiff dogs:

1. Dog typed as *DEA 1*.*1+* by Rapid Vet-H *DEA 1*.*1* (DMS Laboratories, Inc, Flemington, NJ, USA), *Shigeta 1*.*1B+* (Shigeta Animal Pharmaceutical Inc, Oyabe, Japan) and *DEA 1*.*1+* with a polyclonal antiserum (KABB, Sokchosi, South Korea) leading to the production of a monoclonal anti-*Kai 1* antibody;
2. Dog typed as *DEA 1*.*1-* by Rapid Vet-H *DEA 1*.*1*, but *Shigeta 1*.*2B+* and *DEA 1*.*2+* with a polyclonal antiserum (KABB, Sokchosi, South Korea) leading to the production of a monoclonal anti-*Kai 2* antibody.

After washing the anticoagulated canine blood with phosphate buffered saline (PBS, pH 7.2), the samples were adjusted to an RBC count of 1×10^8^ cells/ml. One ml of the RBC suspension was injected intraperitoneally to mice on 17, 10, and 3 days before collection. Under isoflurane anesthesia, the mice were sacrificed and splenic cells were harvested. The cells were fused with mouse myeloma cells (P3X63Ag8.653; ATCC, Manassas, VA, USA) at a ratio of 5:1 with 50% polyethylene glycol (Sigma, St Louis, MO, USA). After isolating hybridoma cells with the hypoxanthine-aminopterin-thymidine medium containing 100 μM hypoxanthine, 0.4 μM aminopterin, and 16 μM thymidine (Sigma), the hybridoma cells causing agglutination specific to the blood used for sensitization were selected in direct hemagglutination assays. The positive hybridoma cells were then inoculated intraperitoneally into pristine-primed female BALB/c mice (n = 6) to yield the ascites containing the monoclonal antibody.

### Purification of antibodies

Ascite was filtered through a 0.45 μm syringe filter and 5-fold diluted with PBS, before passing the fluid through protein L or protein G columns (GenScript, Piscataway, NJ, USA). The column resins were washed 3 times with PBS, and immunoglobulins were eluted with 0.1 M glycine (pH 3.0) buffer and neutralized by addition of 1M Tris-HCl (pH 8.0). The purified monoclonal antibodies were further diluted with PBS and analyzed by bicinchoninic acid (Sigma) and sodium dodecyl sulfate polyacrylamide gel electrophoresis (SDS-PAGE) assays by comparing them to mouse IgG (Abcam, Cambridge, UK).

### Isotype determination of Kai antibodies

ELISA plates coated with goat anti-mouse heavy-chain antibodies (IgG1, IgG2a, IgG2b, IgG3, IgA and IgM) or goat anti-mouse light-chain antibodies (kappa or lambda) were used (Thermo Scientific, Rockford, IL, USA). The purified antibodies against the two canine blood samples were added to each well and the isotypes were then spectrophotometrically determined by measuring the absorbance at 450 nm above 0.2 optical densities.

### Western blotting with canine RBC membrane proteins

Canine RBC membrane proteins were precipitated using a glycoprotein isolation kit (Thermo Scientific) and the protein concentrations were determined (Bio-Rad DC protein assay kit II, Bio-Rad, CA, USA). The lysates containing 50–100 μg of protein were mixed with 4x NuPAGE-LDS sample buffer, and the proteins were separated through NuPAGF^™^ Bis-Tris gels with 1x NuPAGE MES SDS running buffer (Thermo Scientific, Rockford, IL, USA). The gels were electro-transferred onto a Hybond ECL transfer membrane with transfer buffer (25 mM Tris, 250 mM glycine, 20% methanol) at 300 mA for 90 min. To block nonspecific binding sites, the membranes were treated with 5% nonfat dry milk in Tris-buffered saline with Tween 20 (TBST) for 90 min at room temperature (about 22°C). The membranes were incubated overnight with *Kai 1* (1:250 titer) or *Kai 2* (1:100) antibody in TBST containing 3% nonfat dry milk at 4°C. The membranes were washed with TBST three times for 30 min and incubated with goat anti-mouse IgM or IgG HRP conjugated secondary antibody (1:3000) in TBST that contained 3% nonfat dry milk for 90 min at room temperature. After incubation with secondary antibody, the membranes were washed with TBST three times for 30 min at room temperature. The proteins were detected by an enhanced chemiluminescence detection system.

### Affinity chromatography of Kai antibodies

Affinity purification was accomplished by using Affi-Gel affinity chromatography according to manufacturer’s instructions (Bio-Rad, CA, USA). Briefly, 1 mg of anti-*Kai 1* or anti-*Kai 2* antibodies were added into columns filled with Affi-Gel slurry. After overnight incubation at 4°C, the column resins were blocked with 100 mM ethanolamine and washed three times with PBS. Affinity proteins for IgM or IgG were purified using the same columns as those used for antibody purification.

### Comparison of canine blood types between Kai types and commercially available DEA 1 test kit

To determine any relationship between *Kai* types and *DEA 1*, *DEA 1+* or *DEA 1-* samples (total n = 50), typed with Rapid Vet-H *DEA 1* card test (DMS Laboratories, Flemington, NJ, USA), were compared to typing results with *Kai* antibodies.

### Alloantibody detection in transfused dogs

Blood samples from dogs (n = 4) were tested in a tube agglutination assay applying the *anti-Kai 1* and *anti-Kai 2* antibodies as well as for the presence of *anti-Kai 1* and/or *anti-Kai 2* antibodies. In brief, blood samples were obtained from regular blood donors as well as recipient dogs transfused with *Kai 1+* or *Kai 2+* blood. The samples were washed in saline and resuspended to a 2~3% suspension in physiological saline solution. In a 3 ml plastic tube, equal volumes of plasma and RBC suspension were mixed and incubated at 37°C for 15 min. After centrifuging for 1 min at 1000g, the tubes were gently mixed to dislodge the pelleted RBCs. The degree of agglutination strength was macroscopically graded from negative (0) to positive (4+).

## Results

### Production of Kai 1+ and Kai 2+ monoclonal antibodies

Hybridoma cells from a total of 4800 wells were screened for specificity to the canine blood used to sensitize mice. The cell lines were discarded if they agglutinated non-specifically or cross-reacted to all RBCs. We isolated two hybridoma cell lines which produced different, but not cross-reacting, antibodies against the RBCs from the two dogs typed as *DEA 1*.*1* and *DEA 1*.*2*, respectively. We named them *Kai 1* and *Kai 2*, *Kai* meaning ‘dog’ in Korean. The *anti-Kai 1* and *anti-Kai 2* antibodies in the ascitic fluids reacted in RBC agglutination tests up to dilutions of 1:2056 and 1:32, respectively. They did not show any hemolytic activities.

### Isotypes of purified anti-Kai antibodies

**Fig 1:**
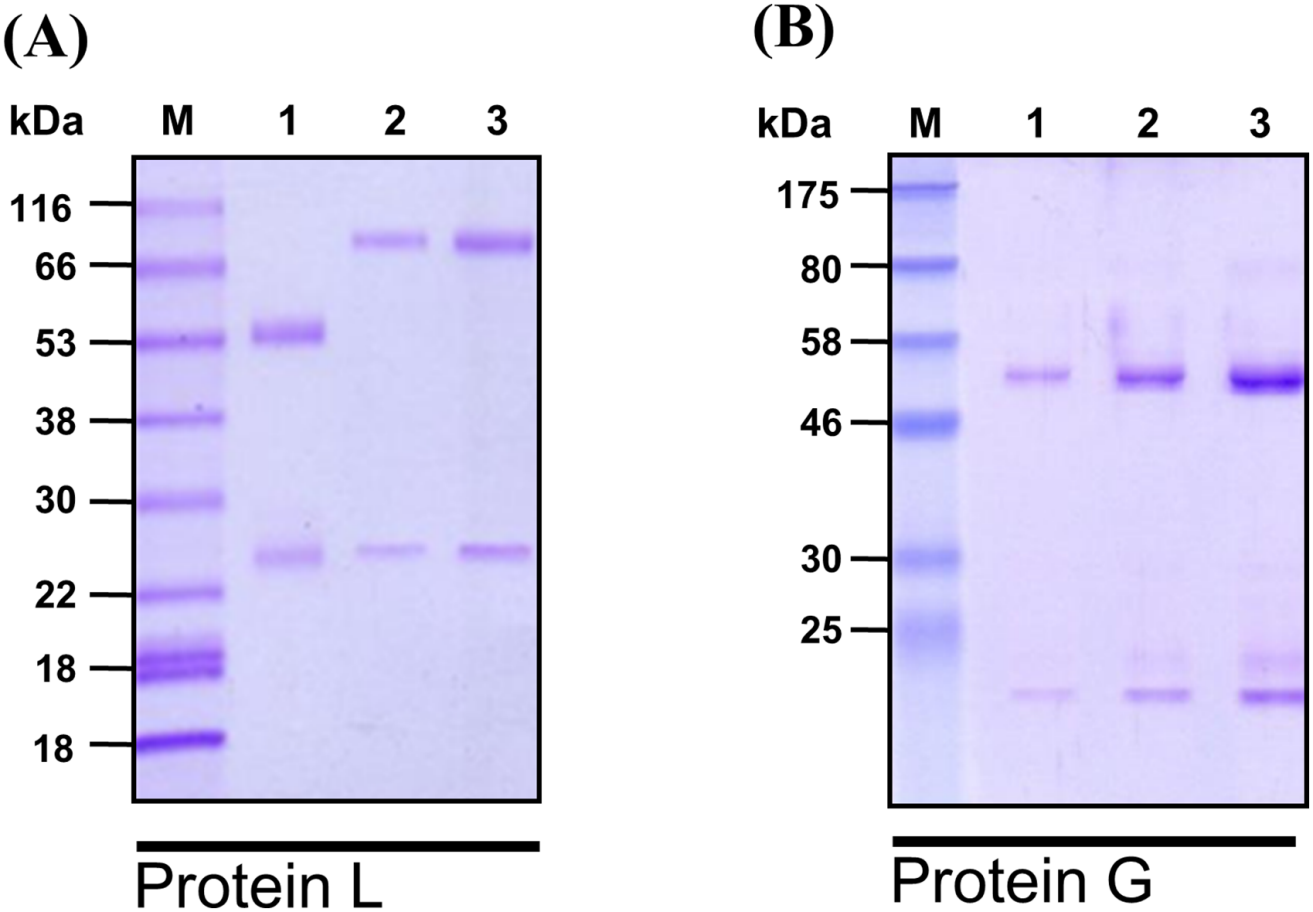
SDS-PAGE (Sodium dodecyl sulfate polyacrylamide gel electrophoresis) of purified (A) *anti-Kai 1* and (B) *anti-Kai 2* monoclonal antibodies. M: Marker (kDa); Lane 1: Normal mouse IgG, Lane 2 and 3: Purified mAb.

Using the Proteins L and G columns and SDS-PAGE, the *anti-Kai 1* immunoglobulins (Ig) were captured by the Protein L column, showing distinct bands at ~80 kDa and ~25 kDa, reflecting the heavy and light chain Ig, different from mouse IgG, thereby suggesting the presence of an IgM (Fig 1A). On the other hand, the *anti-Kai 2* Ig were captured by the Protein G column and revealed ~50 kDa and ~20 kDa bands identical to the heavy and light chain of murine IgG, respectively (Fig 1B).

**Fig 2:**
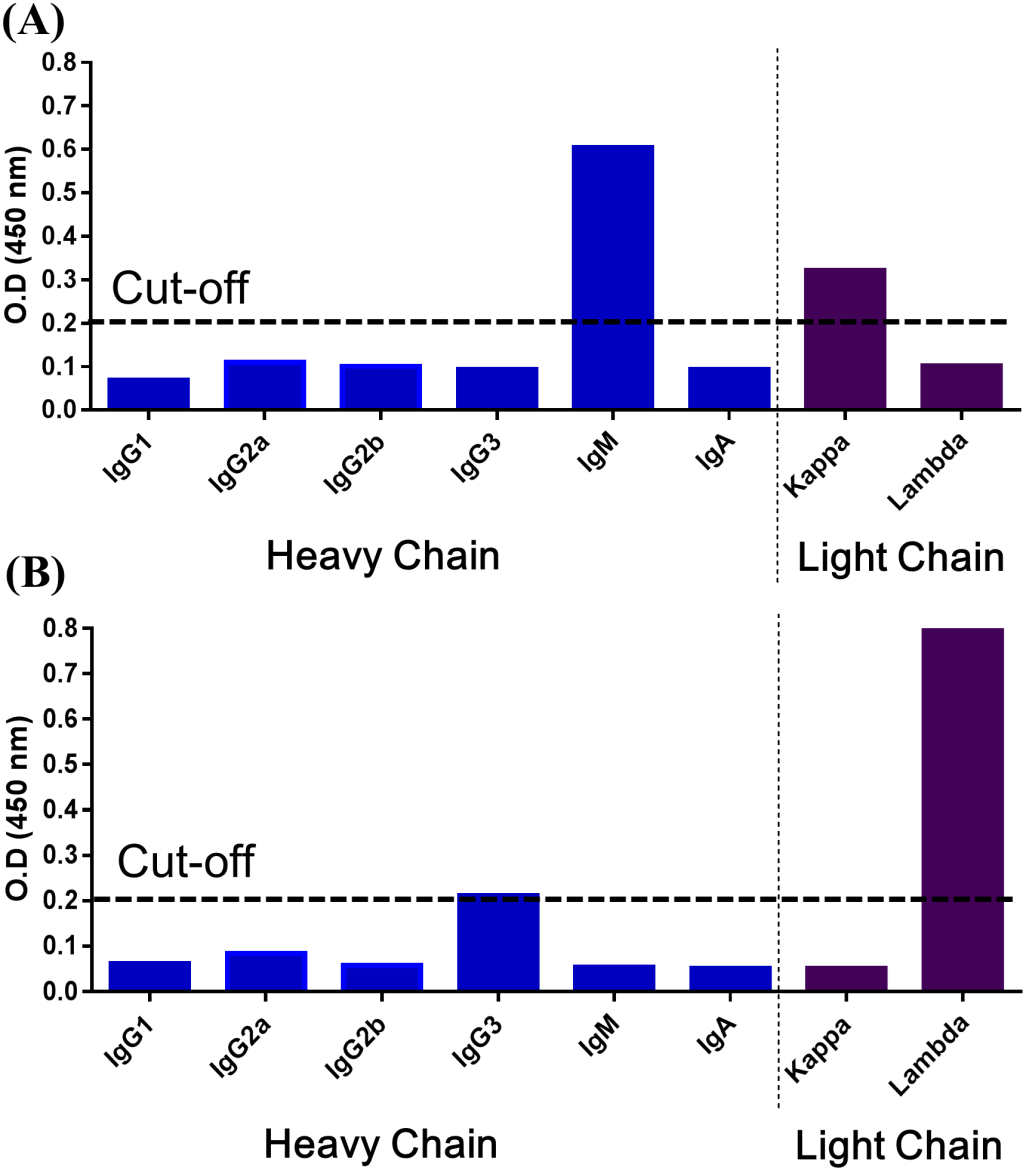
Isotyping of (A) *anti-Kai 1* and (B) *anti-Kai 2* antibodies by ELISA. Results were expressed as optical density (O.D.) values in ELISA, and values >0.2 (horizontal dashed line) were considered positive.

Moreover, by ELISA pre-coated with goat anti-mouse heavy-chain antibodies (IgG1, IgG2a, IgG2b, IgG3, IgA and IgM) or goat anti-mouse light-chain antibodies (kappa or lambda), the *anti-Kai 1* antibody was isotyped as IgM with kappa light chains, whereas *anti-Kai 2* antibody reacted with antibodies against IgG3 heavy and lamda light chain. These results indicate that *anti-Kai 1* and *anti-Kai 2* antibodies belong to subclasses of IgM kappa and IgG lamda, respectively (Fig 2).

### Distribution of Kai types in DEA 1+ and DEA 1- dogs

Utilizing these two mAbs in a conventional tube agglutination typing assay produced either strongly positive (3+/4+) or negative (0) agglutination reactions with canine blood. In a survey of 203 dogs (mostly, Korean Mastiff) from South Korea, 42% *Kai 1+/Kai 2-*, 37% *Kai 1-/Kai 2+* and 20% *Kai 1-/Kai 2-* samples. However, no single blood sample agglutinated with both *anti-Kai 1* and *anti-Kai 2* mAbs.

In addition, when typing 50 of the above dogs with commercially available *DEA 1* test kits (Rapid Vet-H DEA 1) and compared the results with their *Kai* types, there were *DEA 1+* dogs which were *Kai 1+/Kai 1-* or *Kai 1-/Kai 2+*, while the *DEA 1-* dogs could be *Kai 1+/Kai 1-* or *Kai 1-/Kai 2+*, or *Kai 1-/Kai 2-*. This indicates that *DEA 1+* blood can be either *Kai 1+*, *Kai 2+* or entirely *Kai*- (Table 1).

**Table 1:** Comparison of *Kai* and *DEA 1* in 50 dogs.

DEA 1	Kai 1+	Kai 2+	Kai-	Total in % (n)
DEA 1+ (n = 19)	15	4	-	38% (19)
DEA 1- (n = 31)	21	7	3	62% (31)
Total in % (n)	72% (36)	22% (11)	6% (3)	100% (50)

### Identification of the Kai 1 and Kai 2 antigens by Kai mAbs

**Fig 3:**
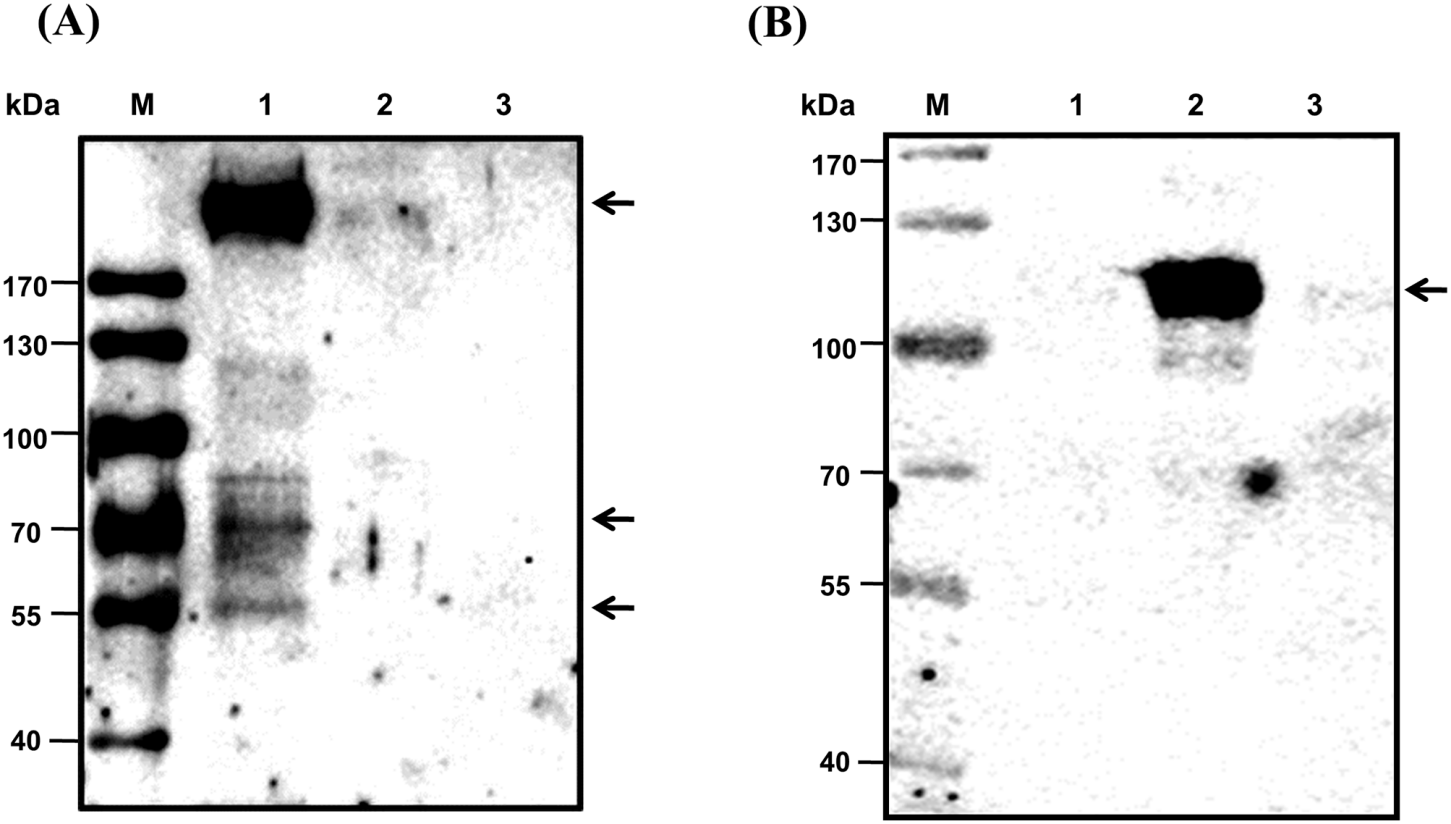
Immunoblot of canine erythrocyte membrane proteins with with (A) *anti-Kai 1* and (B) *anti-Kai 2* monoclonal antibodies. M: Marker (kDa), Lane 1: *Kai 1+/Kai 2-* RBCs, Lane 2: *Kai 1-/Kai 2+* RBCs, Lane 3: *Kai 1-/Kai2-* RBCs.

**Fig 4:**
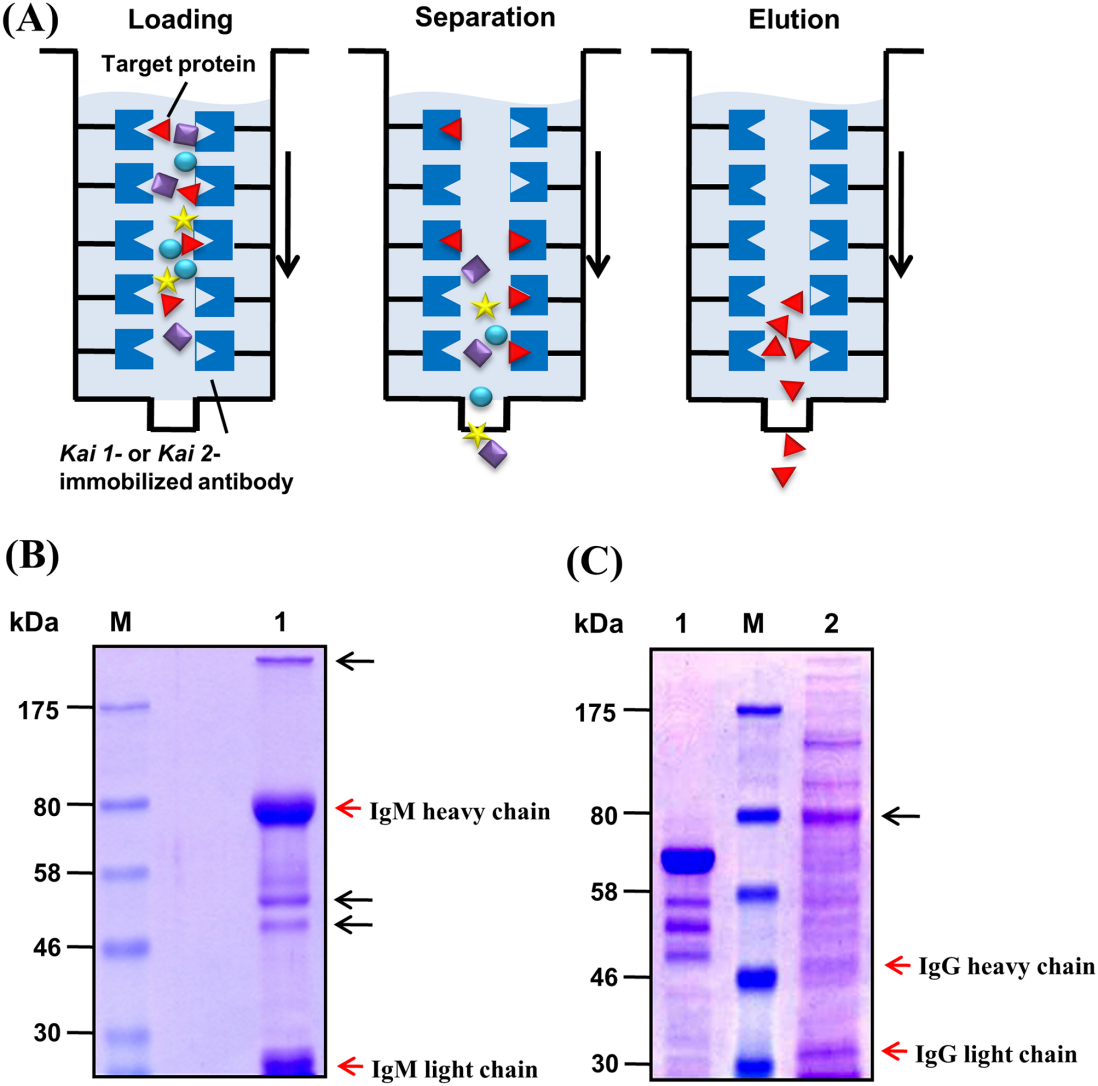
Affinity purification and chromatography of *Kai 1* and *Kai 2* antigens from erythrocyte membrane extracts. (A) Schematic diagram of affinity purification procedure. (B) Membrane proteins from *Kai 1+* RBCs bound with *anti-Kai 1* mAbs and IgM heavy & light chains of *anti-Kai 1* antibody. M: marker (kDa), Lane 1: purified membrane protein (10 μg) from *Kai 1+* RBCs. (C) Membrane proteins *Kai 2+* from RBCs bound with *anti-Kai 2* antibody and IgG heavy & light chains of *Kai 2* antibody. M: marker, Lane 1: bovine serum albumin (2 μg), Lane 2: purified membrane protein (10 μg) from *Kai 2+* blood. Molecular weights are indicated on the left side in kDa.

By immunoblotting under reducing conditions, the *anti-Kai 1* mAb detected specifically a major band >170 kDa and two minor bands of ~55 and ~70 kDa in samples from *Kai 1+* but not *Kai 1-* blood (Fig 3A). With the *anti-Kai 2* mAbs, only one major band between 70 and 130 kDa was seen with samples from *Kai* 2+ dogs (Fig 3B). For more precise identification of protein bands specific to *Kai* 1 or *Kai* 2 antibodies, affinity chromatography was performed and revealed a major band of ~200 kDa and split bands ~50 kDa with the *anti-Kai 1* mAb. On the other hand, *anti-Kai* 2 mAbs bound a prominent band at ~80 kDa (Fig 4).

### Detection of alloantibodies against Kai 1 and Kai 2

**Fig 5:**
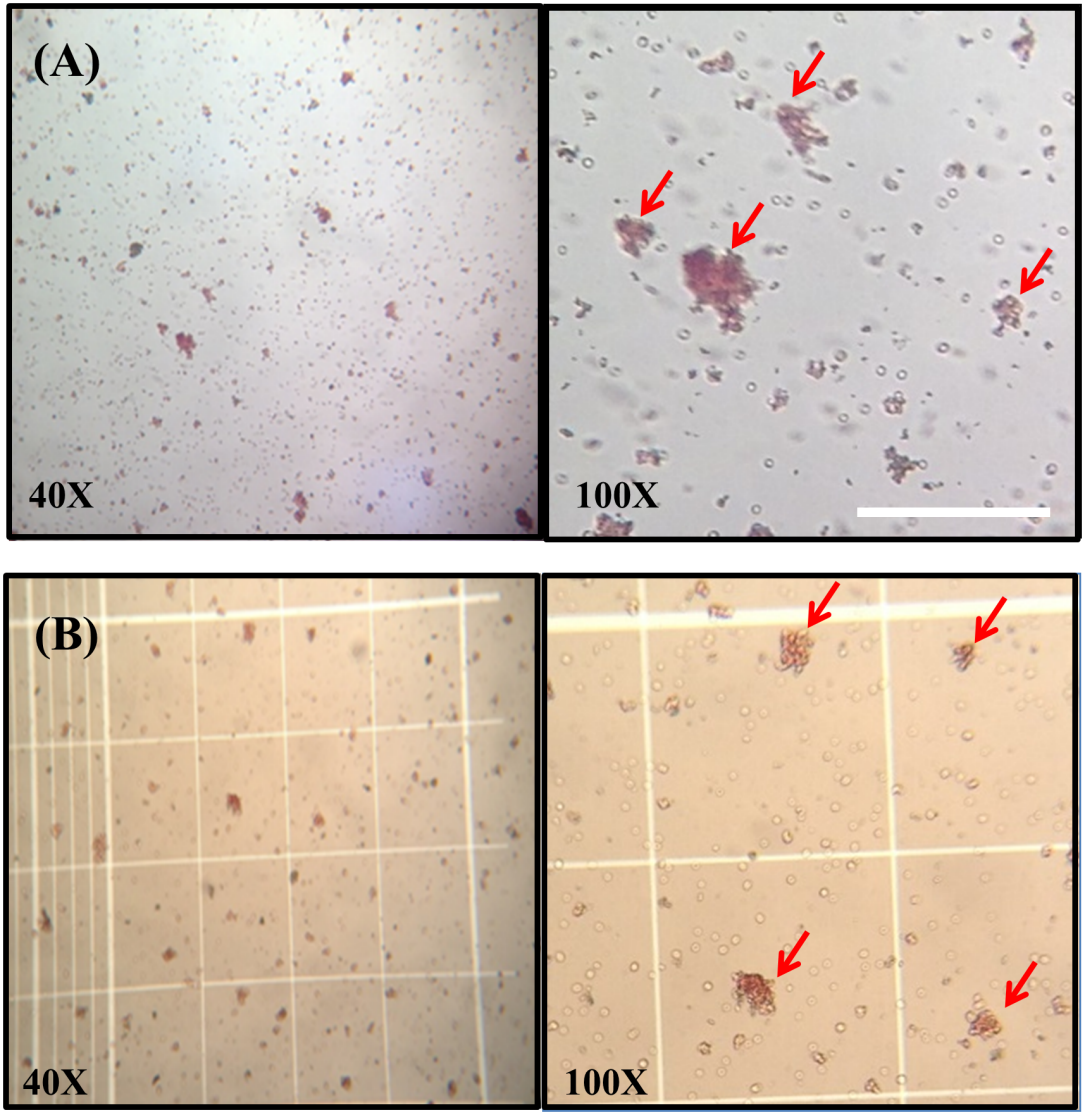
Major crossmatch test incompatibility in two dogs 21 days after receiving *Kai* mismatched transfusions. (A) Microscopic agglutination in case of a *Kai 2-/DEA 1+* dog receiving *Kai 2+/DEA 1+* blood. (B) Microscopic agglutination in case of a *Kai 1-/DEA 1+* dog receiving *Kai 1+/DEA 1+* blood. Bar = 200 μm.

Based upon major crossmatch tests with *Kai 1* and *Kai 2* mismatched blood, pre-transfusion compatibility testing did not show any agglutination reactions. However, when crossmatching plasma from previously transfused dogs ~21 days after transfusion, +1 to +3 agglutination reactions were observed when the recipient was *Kai 1-* or *Kai 2-*, and the donor was *Kai 1+* or *Kai 2+* (Table 2). Those recipients were *DEA 1+* but not typed for other blood groups. Many blood clots were microscopically seen in a *Kai* 2+/*DEA 1-* (Fig 5A) and a *Kai* 1+/*DEA 1+* blood-transfused dog (Fig 5B).

**Table 2:** Major cross-match results in the transfused dogs related to *DEA 1* and *Kai*.

			Day 0	Day 21
Dog	Recipient blood type	Donor blood type	Cross-Match Results	Agglutination Strength
1	Kai 1+, DEA 1+	Kai 2+, DEA 1+	Compatible	1+
2	Kai 2+, DEA 1+	Kai 1+, DEA 1+	Compatible	1+
3	Kai 1+	Kai 2+	Compatible	3+
4	Kai 2+	Kai 1+	Compatible	3+

## Discussion

While based upon experimental and clinically mismatched transfusions more than a dozen blood groups have been described in dogs, the nature of these canine alloantibodies and RBC antigens have remained mostly elusive. We produced two distinct monoclonal antibodies which are strongly agglutinating and bind two apparently new dog erythrocyte antigens named *Kai 1* (200 and 50 kDa) and *Kai 2* (80 kDa). The *Kai 1* and *Kai 2* antibodies were isotyped as IgM kappa and IgG3 lamda, respectively, and identified two different dog erythrocyte membrane proteins of 200 kDa and 80 kDa, respectively. We show that *Kai 1-* and/or *Kai 2-* dogs developed *anti-Kai 1* and *anti-Kai 2* alloantibodies, respectively, when transfused with mismatched blood demonstrating the clinical importance of these *Kai* blood groups.

In veterinary transfusion medicine, hybridoma techniques have thus far only been applied to produce mAbs against canine *DEA 1*.*1* (later named *DEA 1*) and *DEA 3* antigens, feline *A* and *B* antigens and the equine Ca antigen [11–14]. In our attempt to produce mAbs against *DEA 1*.*1* and *DEA 1*.*2* antibodies reported here, we generated and characterized two mAbs against canine erythrocyte antigens, named *Kai 1* and *Kai 2*. The *anti-Kai 1* mAb isotyped by ELISA as IgM kappa and was a much stronger agglutinin (1:2056) compared to the *anti-Kai 2* IgG3 lamda (1:32) against the *Kai+* erythrocytes. Indeed, IgG mAbs can also effectively agglutinate erythrocytes [15], are more stable than IgM in accelerated storage tests, and frequently exhibit higher affinity than IgM [16]. The two *anti-DEA 1* mAbs used in typing kits are either IgM (DMS laboratories Inc., Tempe, AZ) or IgG (Alvedia, Lyon, France) [17]. The *anti-DEA 3* mAb was not characterized and is currently not available for typing. And all other alloantibodies for typing dogs are polyclonal antibodies generated accidently or experimentally by sensitizing dogs by RBC transfusions [3].

The generated *anti-Kai 1* and *anti-Kai 2* alloantibodies cause strong agglutination reactions in the tube, as shown here, and gel column tests used in our recent typing survey [18]. While both techniques readily differentiate *Kai+* and *Kai-* RBCs; the gel column technique is simpler to read and photographically capture for archiving, but requires a special centrifuge. Thus, these *Kai* mAbs are suitable as typing reagents in clinical settings. In the surveys reported here from Korea (with mostly Mastiffs as blood donors typed) and previously from North America [18], *Kai 1+/Kai 2-* (42% in Korea; 94% in North America) dogs were commonly found, while *Kai 1-/Kai 2+* (37% in Korea; 1% in USA) and *Kai 1-/Kai 2-* (20% in Korea; 5% in North America) were less frequently observed. The variation in the two surveys regarding the prevalence of each *Kai* type may be related to differences in geographical areas and breeds typed as well as the relatively small size of the surveys. Noteworthy, neither survey detected any dogs which were *Kai 1+/Kai 2+* suggesting the two antigens may not be coexisting in any dogs.

When comparing the *Kai* typing results and *DEA 1* with available mAbs from the small survey of 50 dogs reported here and >500 dogs from North America reported recently [18], it is evident that *Kai 1+* or *Kai 2+* were more frequently *DEA 1+*, but could also be rarely *DEA 1-*. Similarly, *Kai 1-* and/or *Kai 2-* dogs could be *DEA 1+*. Thus, the *anti-Kai* and *anti-DEA 1* mAbs do not recognize the same blood types in dogs. This was surprising as the *Kai* mAbs were generated against supposedly against *DEA 1*.*1+* and *DEA 1*.*2+* blood. The blood initially used for sensitizing mice was typed with reagents from polyclonal antisera (KABB) and *Shigeta DEA 1*.*1+* (*1*.*1B+*) and as *DEA 1*.*2+* (*1*.*2B+*). Unfortunately, neither the original dogs used for sensitization nor the *Shigeta* typing reagents were available to further compare and investigate these apparent discrepancies. Furthermore, in our previously reported survey, a relationship between *DEA 3*, *DEA 4* and *DEA 7* as well as *Dal* could not be identified [18]. Also, it has become evident that the *DEA 1* blood group system is composed of varied *DEA 1* types and not *DEA 1*.*1* and *1*.*2* (see below)

Despite considerable research efforts over the past decades, the nature of the canine blood group antigens remains largely unknown. Some *DEAs* have been assigned specific molecular weights, but those studies have not progressed or confirmed by others. In affinity immunochromatographic assays reported here, the *anti-Kai 1* mAb identified a protein of ~200 kDa and ~50 kDa (two split bands), while the *anti-Kai 2* mAb detected a protein of ~80 kDa. These proteins have not been further purified and characterized by sequencing. Interestingly, the *anti-Kai 1* and *anti-Kai 2* binding proteins of 200/50 kDa and 80 kDa concur with earlier protein studies for *DEA 1*.*1* [11] and *DEA 1*.*2* [19], respectively, despite the above discordant *Kai* and *DEA 1* blood typing results. However, it would be highly unusual to have two different proteins for *DEA 1*.*1* and *DEA 1*.*2* be part of the same blood group system. Recent studies using the available *anti-DEA 1* mAbs failed to confirm these earlier protein studies on *DEA 1* [17]. In the meantime, further flow cytometric studies with currently available *anti-DEA 1* mAbs have shown that *DEA 1*.*1* and *DEA 1*.*2* are the same antigen and did not correlate to the variations in the expression of *DEA 1* with *DEA 1-* and weakly, moderately and strongly *DEA 1+* dogs [17]. Furthermore, a preliminary genome wide association study found a single nucleotide polymorphism in a region on chromosome CFA27 for *DEA 1* [20]. Further biochemical and molecular genetic studies will be needed to characterize and clarify the relationship of the proteins found by immunoblotting and reactions with various monoclonal and polyclonal antibodies. Maybe the *Kai 1* and *Kai 2* may correspond to the originally described erythrocyte proteins *DEA 1*.*1* and *DEA 1*.*2* and the currently used *anti-DEA 1* mAbs recognize another RBC antigen.

While originally debated dogs generally do not have naturally occurring alloantibodies to known blood groups [4] and pregnancy does not induce alloantibodies due to their endoepithelial placenta which is impermeable to proteins [21]. Similarly, we show here as well in the previous study that *Kai 1-* and/or *Kai 2-* dogs do not have *anti-Kai 1* or *anti-Kai 2* alloantibodies in their plasma, respectively. However, when evaluating the plasma from a small number of previously transfused *DEA 1+* dogs, we discovered *anti-Kai 1* and/or *anti-Kai 2* alloantibodies in plasma from those typed as *Kai 1-* and/or *Kai 2-*. This documents the antigenic nature of both of these erythrocyte membrane proteins and thereby making *Kai 1* and *Kai 2* qualify as new independent blood group systems.

However, the clinical importance of developing *anti-Kai 1* and/or *anti-Kai 2* alloantibodies still needs to be determined by showing accidental or experimental acute hemolytic transfusion reactions in previously transfused *Kai 1-* and/or *Kai 2-* dogs receiving a second transfusion more than four days apart. The development of post-transfusion alloantibodies has been shown clinically in a few surveys [3,22], but only in rare occasions the causative blood type mismatch such as *DEA 1*.*1*, *DEA 4* and *Dal*, was found. As the polyclonal *anti-Dal* [23,24] and monoclonal *anti-Kai* reagents become more available for typing and recipients can be type-matched beyond the currently recommended *DEA 1*, sensitization and transfusion reactions can be further avoided. In the meantime, the current recommendation of transfusing *DEA 1-* or *DEA 1* matched blood at time of first transfusion initially and also crossmatch compatible blood for any subsequent transfusion (>4 days) may be extended to including typing for *Kai* dogs with incompatible crossmatch to find more readily compatible donors which had been also typed from *Kai* [18]. It should be noted that despite 36 blood group system and 352 antigens in humans [25], routine typing remains fairly restricted to ABO and Rh (and possibly Kell) unless in special circumstances or when the patient is found to be crossmatch incompatible [26].

In conclusion, two blood groups named *Kai 1* and *Kai 2* were identified and characterized with mAbs which differ based upon typing surveys from *DEA 1* and other known and tested blood types. Affinity immunoblots identified distinctly different erythrocyte membranes which coincide with earlier described *DEA 1*.*1* and *DEA 1*.*2* antigen sizes. And while the antigenicity of these antigens was shown in crossmatch tests, the clinical importance of these induced alloantibodies needs to be still determined. Blood typing beyond *DEA 1* typing including *Kai* and *Dal* typing may be helpful to explain blood type incompatibilities in previously transfused dogs and donors.

## Supporting information

S1 File Owner agreement. (PDF)

S2 File NC3Rs ARRIVE guidelines checklist. (PDF)
