# Supplementary material for: Kai 1 and Kai 2: Characterization of these dog erythrocyte antigens by monoclonal antibodies
Source: PLoS One. 2017 Jun 29;12(6):e0179932. doi: 10.1371/journal.pone.0179932 (PMC5491067; doi:10.1371/journal.pone.0179932)
Supplement: S1 File — (PDF) [file pone.0179932.s001.PDF]

동의서

(Owner agreement)

진료 중 채혈된 혈액샘플에 대한 분석결과에 대하여

실험적인 사용을 허락합니다.

(I allow for experimental use of the laboratory results of blood samples collected during medical treatment)

날짜 (DATE): 2017. 4. 20

전화번호: 010 - 9434 - 3747

이름 (NAME): 윤 옥 경

회신 팩스 번호 (Returning Fax Number): 053-770-2288

동의서

(Owner agreement)

진료 중 채혈된 혈액샘플에 대한 분석결과에 대하여

실험적인 사용을 허락합니다.

(I allow for experimental use of the laboratory results of blood

samples collected during medical treatment)

날짜 (DATE): 2017. 4. 20

전화번호: 010 – 9645-5150

이름 (NAME): 주 명 찬

회신 팩스 번호 (Returning Fax Number): 053-770-2288

동의서

(Owner agreement)

진료 중 채혈된 혈액샘플에 대한 분석결과에 대하여

실험적인 사용을 허락합니다.

(I allow for experimental use of the laboratory results of blood samples collected during medical treatment)

날짜 (DATE): 2017. 4. 20

전화번호: 010 - 3016 - 4425

이름 (NAME): 이 장 춘

회신 팩스 번호 (Returning Fax Number): 053-770-2288

동의서

(Owner agreement)

진료 중 채혈된 혈액샘플에 대한 분석결과에 대하여

실험적인 사용을 허락합니다.

(I allow for experimental use of the laboratory results of blood samples collected during medical treatment)

날짜 (DATE): 2017. 4. 20

전화번호: 010 - 7383 - 0967

이름 (NAME): 구 상 민

회신 팩스 번호 (Returning Fax Number): 053-770-2288

2017-APR-21 09:59 From:HANMI 0313715119 To:70537686340 P.3/4

동의서

(Owner agreement)

진료 중 채혈된 혈액샘플에 대한 분석결과에 대하여

실험적인 사용을 허락합니다.

(I allow for experimental use of the laboratory results of blood samples collected during medical treatment)

날짜 (DATE): 2017. 04. 20

전화번호: 033-631-8575

이름 (NAME): Director Kim Joo Kyung

Korea Animal Blood Bank

회신 팩스 번호 (Returning Fax Number): 053-770-2288
